# Decision tree–based classifier in providing telehealth service

**DOI:** 10.1186/s12911-019-0825-9

**Published:** 2019-05-30

**Authors:** Ching-Chin Chern, Yu-Jen Chen, Bo Hsiao

**Affiliations:** 1 Department of Information Management, National Taiwan University, 50, Lane 144, Sec. 4, Keelung Road, Taipei, 106 Taiwan; 2 Department of Information Management, Chang Jung Christian University, No.1,Changda Rd., Gueiren District, Tainan City, 71101 Taiwan

**Keywords:** Telehealth service, Decision tree, Data mining, Data preprocessing

## Abstract

**Background:**

Although previous research showed that telehealth services can reduce the misuse of resources and urban–rural disparities, most healthcare insurers do not include telehealth services in their health insurance schemes. Therefore, no target variable exists for the classification approaches to learn from or train with. The problem of identifying the potential recipients of telehealth services when introducing telehealth services into health welfare or health insurance schemes becomes an unsupervised classification problem without a target variable.

**Methods:**

We propose a HDTTCA approach, which is a systematic approach (the main process of HDTTCA involves (1) data set preprocessing, (2) decision tree model building, and (3) predicting and explaining of the most important attributes in the data set for patients who qualify for telehealth service) to identify those who are eligible for telehealth services.

**Results:**

This work uses data from the NHIRD provided by the NHIA in Taiwan in 2012 as our research scope, which consist of 55,389 distinct hospitals and 653,209 distinct patients with 15,882,153 outpatient and 135,775 inpatient records. After HDTTCA produces the final version of the decision tree, the rules can be used to assign the values of the target variables in the entire NHIRD. Our data indicate that 3.56% (23,262 out of 653,209) of the patients are eligible for telehealth services in 2012. This study verifies the efficiency and validity of HDTTCA by using a large data set from the NHI of Taiwan.

**Conclusion:**

This study conducts a series of experiments 30 times to compare the HDTTCA results with the logistic regression findings by measuring their average performance and determining which model addresses the telehealth patient classification problem better. Four important metrics are used to compare the results. In terms of sensitivity, the decision trees generated by HDTTCA and the logistic regression model are on equal grounds. In terms of accuracy, specificity, and precision, the decision tree generated by HDTTCA provides a better performance than that of the logistic regression model. When HDTTCA is applied, the decision tree model generates a competitive performance and provides clear, easily understandable rules. Therefore, HDTTCA is a suitable choice in solving telehealth service classification problems.

## Background

The concept of telehealth, which first appeared in the 1900s when physicians began discussing diseases by telephone and has evolved to such a sophisticated level of performing robotic surgery, regardless of the geographical restrictions, is possible at present [19]. Telehealth uses electronic information and communication technology to deliver health and medical information and services over large and small distances [11, 19]. The U.S. Health Resources and Services Administration defines telehealth as “the use of electronic information and telecommunications technologies to support long-distance clinical health care, patient and professional health-related education, public health, and health administration” [7]. For chronically ill patients and people with disability who require frequent updates of health parameters, telehealth services can provide convenience, mobility, and ease of use. As the aging population and people with disability increase, teleassistance and telemonitoring platforms play increasingly significant roles in delivering efficient and low-cost remote care in assisted living environments [22].

The emergence of wireless technologies and the advancements in on-body sensor design can facilitate changes in the conventional healthcare system by replacing it with wearable healthcare systems centered on individuals [21]. For example, devices and techniques in monitoring blood pressure, blood glucose level, cardiac activity, and respiratory activity are recent advances in noninvasive monitoring technologies for chronic disease management. Patients can improve or maintain their health states by using telecommunication and information technology without the need to schedule in-person healthcare visits. However, designing a telemetry system for health monitoring is complicated and expensive, and insurance providers must carefully consider and calculate who will benefit most from it.

A new concept called elderly welfare, which incorporates health welfare and the development of a telehealth system for the aging population, also has emerged. The telecare industry has expanded worldwide. Many countries, such as Japan, the United Kingdom, the United States, and Canada, have developed long-term care assistance policies to utilize telehealth systems [5, 6, 18]. Previous studies [2, 10, 13] summarized the benefits of adopting telehealth systems for three stakeholders namely, (1) cost-saving for patients and health care facilities, (2) far-reaching care for patients, (3) reduced delays in medical treatment for chronic patients, (4) reductions in healthcare facility admission rates and duration of outpatient visits, and (5) improved quality of life for countries as a whole.

If patients must pay their own expenses for telehealth services without insurance reimbursement, then extremely few patients will have motivation to use these services [12, 15]. However, an elderly patient who lives alone in a remote village may spend more than 8 h in transit to see a physician in a healthcare facility, which can actually worsen a patient’s chronic disease condition. For example, patients with diabetes or hypertension may be unaware of an abnormality and miss the crucial time to see a physician. Telehealth services can reduce the urban–rural gap in allowing for patients in remote areas to medical resources without long transport time [12, 15].

When Taiwan introduced its National Health Insurance (NHI) plan in 1995, the Department of Health also introduced a pilot project in providing telehealth services [12]. Nevertheless, a review of the government plan in 2013 [16] showed that the number of cumulative applicants is only 9606 with up to 343,000 times of services at the end of 2011. Researchers have identified several main reasons why patients in Taiwan seldom use telehealth services. First, patients are willing to pay less than 1000 New Taiwan Dollars (NTD) monthly on the average for a telehealth service, but renting remote physiological monitoring equipment costs at least 3000 NTD monthly, excluding service fees [12]. Second, outpatients prefer receiving medical advice in person, and they are not accustomed to use the telehealth services [20]. Third, because telehealth services are excluded in NHI coverage, paying for prevention is not an attractive option compared with the deductibles in medical treatment [6]. Despite these reasons, researchers [3, 4, 9, 11] still found numerous social benefits of using telehealth services, including reductions in hospitalization frequency, healthcare facility medical costs, and caregiver’s burden.

Given that health insurance policy has not officially recognized telehealth services as an efficient treatment, we have no information to compute the cost and benefit of using them if they are reimbursed by health insurances. All patients must be classified into two groups, namely, “need telehealth service” and “do not need telehealth service” which will be a time-consuming task without computer aid. Thus, a proper classification algorithm developed with telehealth experts’ assistance is necessary. Among the many classification algorithms, decision trees are the most suitable one because they are simpler to understand and interpret than association rules or logistic regression. Decision trees also require a simple data preparation stage and can handle categorical data.

This study aims to address the problem of identifying the patients who are the best candidates in receiving telehealth services subsidized by health insurance reimbursements. Specifically, patients with certain chronic diseases can benefit from noninvasive monitoring devices such as those evaluating blood pressure, blood glucose levels, and cardiac activity [21]. However, designing a telehealth system with professional health care staff to operate these noninvasive devices is complicated and costly. To prevent overburdening the telehealth system before insurers implement a telehealth reimbursement policy, researchers must identify the best qualified patients in receiving telehealth services to ensure that the neediest patients are assisted, instead of simply those who able can pay for them.

Although previous research showed that telehealth services can reduce the misuse of resources and urban–rural disparities [2], most healthcare insurers do not include telehealth services in their health insurance schemes [6, 12]. Therefore, no target variable exists for the classification approaches to learn from or train with. Thus, the problem of identifying the potential recipients of telehealth services when introducing telehealth services into health welfare or health insurance schemes becomes an unsupervised classification problem without a target variable.

The first challenge of this study is to generate the target variable for the unsupervised telehealth classification problem. The type of target variable (interval, ordinal, or nominal) determines which data-mining techniques can be used. In classifying patients into recipients and nonrecipients, the target variable is generally the patient’s status (e.g., unqualified or qualified). The target variable can also be defined according to different classes in matching their various meanings. For example, we can classify all patients into several classes, such as “necessary”, “maybe necessary in the long term”, and “unnecessary”. However, having many classes, leads to frequent misclassification, because the individuality of each class becomes diluted, which results in misclassification for similar classes. This condition also can lead to an overfitting problem caused by the excess number of predictor variables for a multiclass target variable but insufficient data points. Consequently, this study limits the number of target variable’s classes into two as binary. Thus, the target variable can be transformed into a 0/1 code and the telehealth service classification problem can be applied easily in many data-mining techniques, including decision trees and logistic regression.

The second challenge of this study involves generating the required information from the existing attributes for the insurance providers to determine whether the applicant is a suitable recipient of telehealth services. In a telehealth classification problem, the attributes are the patients’ personal and outpatient information they provided when they submit telehealth service applications. However, applying these attributes directly from the data set to a data classification technique may be inappropriate. For example, the decision tree–building algorithm does not handle numeric attributes uniformly. When applying the numeric attributes to generate the decision tree, numeric attributes may be used more than once with different thresholds. Some important attributes are excluded in the data set; thus, this attributed need to be derived from other attributes. For example, the patient’s traveling distance or transportation time to the hospital is generally not included in the healthcare data set. Thus, these data need to be generated. Solving this challenge can ensure that insurance providers receive the patients’ detailed medical-related data that can be used to generate the telehealth service classifier and applied directly in the classifier in determining the status of an applicant.

Third challenge is building a classifier to solve the problem of identifying candidates in receiving health insurance reimbursement for telehealth services. In this study, we choose decision trees in generating the classifier because the rules they generate are simple to interpret, such that the results can be easily understood for both medical professionals and patients. Constructing a decision tree–based classifier involves three main steps, namely, variable selection, node splitting, and tree pruning [17]. Generally, researchers use entropy and information gains for the first step and then obtain the local maximum information by splitting the data according to a variable. Given that this method requires the data to be categorical, researchers have developed various methods for interval data, such as ID3, C4.5 and CART. Building a decision tree–based classifier also involves applying appropriate feature selection and feature extraction to enhance classification performance. Feature selection is a process of selecting representative attributes; meanwhile, feature extraction transforms the original attributes to some other forms in decreasing the dimensions of the data set. For the current study, we must determine which node-splitting approach together with feature selection and feature extraction, is most suitable to build the classifier. After splitting nodes to generate a tree, the next step is pruning the tree if it has extremely many levels or nodes in avoiding an overfitting problem. Two pruning approaches have been developed, as follows [14]; pre-pruning stops the tree from growing before the entire training data set is classified and post-pruning prunes the tree after the decision tree is finished. For the current study, we must determine which pruning approach is most suitable to build the classifier.

Finally, a fourth challenge is selecting a validation method. Validation is the process of assessing how well the classification models perform against the validation data (real data) by verifying whether the models’ misclassification rates meet the established requirements. The validation techniques consider the probability of the worst-case scenario, wherein a model’s complexity is high. For example, the widely used *k*-fold validation technique divides a data set into *k* subsets and takes *k –* 1 subsets as the training data, with the remainder as the validation data set. Then, the model is trained for *k* times, and each iteration uses the subset *i* one at a time. However, the problem considered herein has a relatively small training data set for the experts to classify the patients as candidates in receiving telehealth services. Given that the training data set is extremely small, we will not split (*k*-fold validation) or cross-validate the training set in the validation step. We need to develop a new validation method suitable for an unsupervised classification problem with an extremely small training set and an exceedingly large test data set.

In summary, this study aims to solve the unsupervised classification problem of identifying the patients who are the best candidates in receiving telehealth services. Four challenges, such as (1) generating the target variable, (2) generating the needed information from the existing attributes, (3) building a classifier, and (4), selecting a validation method, are addressed.

## Methods

**Fig. 1:**
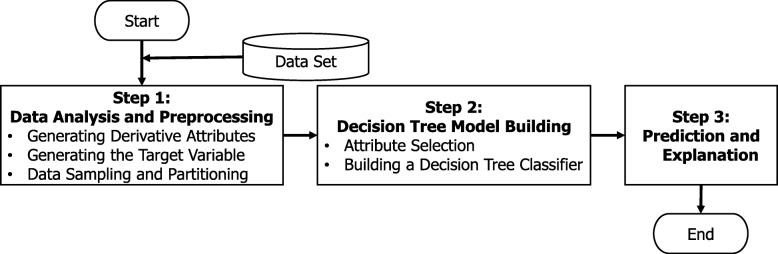
Flow Chart of HDTTCA

To classify candidates to receive telehealth services through health insurance reimbursements, we propose a new decision tree approach, that is, heuristic decision tree telehealth classification approach (HDTTCA), which consists of three major steps, namely, (1) data analysis and preprocessing, (2) decision tree model building, and (3) prediction and explanation, as shown in Fig. 1.

As mentioned before, four challenges are addressed in HDTTCA: step 1 tackles challenges 1, 2 and 4, while step 2 tackles challenges 3 and 4. Finally, in step 3, HDTTCA predicts and explains incoming data by using the decision tree classification model that was chosen previously in step 2. In other words, after building the decision tree model, we use this model to predict the applicability of telehealth services. In the following subsection, we explain the details of steps 1 and 2 and then clarify the time complexity of HDTTCA.

### Step 1: data analysis and preprocessing

As discussed previously, the target variable and some important attributes are excluded in the original data set. Therefore, HDTTCA first needs to derive some attributes from the current data set and then used them in determining the value of target variable. To validate the performance of the decision tree classification model, HDTTCA divides the data set into several subsets.

#### Step 1.1 generating derivative attributes

Given that the raw data containing the critical attributes are often collected from different sources, these data should be integrated into a single data set first. We focus on the two primary actors involved in healthcare activity, namely, patients and hospitals. The patient-related data sets describe the information about those who have seen physicians and contain two types of information, namely, basic information (patients’ important attributes, including gender, age, address, and health history) and clinical information (all medical activities the patients received, including medical treatments and physician visits). We retain only the important attributes of patient-related data sets for telehealth services, such as the hospital where a patient seeks treatment, the code for the international classification of diseases, and the number of days of prescription. The hospital-related data sets describe the information about hospitals that patients visit to see their physicians. For our purposes, we only need the hospital’s location and size.

To reduce the numbers of age categories and balance the percentages among them, we use an attribute, that is, age group (*F*_*Age*_), to represent the age of ≤30 years as young, 30 years ≤ age ≤ 70 years as middle-aged and age of ≥70 years as elderly. Similarly, we transcode the monthly insurance amount into an attribute, that is, insurance level (*F*_*IL*_), which is categorized as low, middle, and high with the suggested percentages of 20, 60 and 20%, respectively. We also mark the situations when patients are not required to pay the copayments with an attribute, that is, copayment exemptions (*F*_*CEM*_) mark as Y and N.

We summarize the number of times in a year that each patient visits a hospital (outpatient) or is hospitalized (inpatient) into op_time and ip_time, respectively. We use an attribute, that is, outpatient frequency (*F*_*op*_) to categorized op_time into none when its value is 0, low for 1 ≤ op_time ≤ 12, middle for 13 ≤ op_time ≤ 36, and high when its value ≥37. We also denote an attribute, that is, inpatient frequency (*F*_*ip*_), as 0 for ip_time = 0, 1 for ip_time = 1, 2 for ip_time = 2, and 3+ for ip_time ≥ 3.

Some diseases are inapplicable for telehealth services (e.g., a car accident victim that went to the emergency room for treatment for the injury and then rests in chronic care for rehabilitation). Therefore, we differentiate the total number of days that a patient uses an emergency bed (EB day) and a chronic bed (CB day). We use an attribute, that is, chronic bed rate (*F*_*CBR*_), which is the number of CB days divided by EB day + CB day, to distinguish those whose symptoms cannot be helped by telehealth services and eliminate the patients with *F*_*CBR*_ = 0. We also summarize the number of drug prescription days as drug day and the total amount of the medical fees as total amount.

In previous studies, the critical influence factors for adopting telehealth services include the patient’s traveling distance or transportation time to the hospital, health status, and financial status [5, 6, 20]. However, these attributes are not recorded directly in the data sets; thus, they need to be derived from existing attributes. Given that telehealth services are more beneficial for patients who live further away from the hospitals, transportation time should be determined when a patient travels from home to the hospital. We can generate the distance of a patient travelling from home to hospital [1], that is, distance (*F*_*Dis*_), by combining the zip codes of the hospital location and the patient’s residence location and the assistance of Google Maps. We use the great-circle distance to estimate the shortest distance between two points on the surface of a sphere, which is calculated as follows:

$$ {F}_{Dis}=r\ {\cos}^{-1}\left[\sin\ {\upvarphi}_1\times \sin\ {\upvarphi}_2+\cos\ {\upvarphi}_1\times \cos\ {\upvarphi}_2\times \cos |\ {\uplambda}_1-{\uplambda}_2|\right]\ \mathrm{km} $$

where (φ_1_, λ_1_) and (φ_2_, λ_2_) denote the latitudes and longitudes of points 1 and 2 (in radians), respectively; and *r* is the mean earth radius (approximately 6371 km). For example, the longitude and latitude of the zip codes 100 and 700 are (121.5199, 25.0324) and (120.1929, 22.99594), respectively. Therefore, the distance between the two points is calculated as follows:

$$ {F}_{Dis}=6371\times {\cos}^{-1}\left[\ \sin \left(25.0324\uppi /180\right)\times \sin \left(22.9959\uppi /180\right)+\cos \left(25.0324\uppi /180\right)\times \cos \left(22.9959\uppi /180\right)\times \cos \left(121.5199-120.1929\right)\uppi /180\right]=263.5161\ \mathrm{km} $$

Telehealth services are beneficial for the patients with chronic diseases because the administration period is > 7 days. We create a special attribute, that is, drug duration (*F*_*DD*_) to record whether a drug is administered for an extended period of time. We use the attribute economic priority (*F*_*Eco*_), to distinguish patients with special conditions, such as low income or disability. Telehealth services are mostly needed by patients living in rural areas, even if their traveling distances to the hospitals are shorter than those of the other patients. Given that remote area is undefined, we use an attribute, that is, remoteness (*F*_*R*_), to distinguish patients residing in rural areas by changing their addresses.

As mentioned previously, telehealth equipment can monitor only some physiological values, such as blood pressure, blood glucose level, and cardiac activity, at present [21]. Thus, telehealth equipment is mostly useful for target diseases, such as diabetes, hypertension, and hyperlipidemia. We highlight the disease codes in a special attribute, that is, target disease (*F*_*TD*_), with Y indicating suitability and N indicating unsuitability for telehealth services, respectively. Another way to mark the potential telehealth users is differentiating the treatment that a patient receives. We create an attribute, that is, target treatment (*F*_*TT*_), to record the specific treatments for diabetes, hypertension, and hyperlipidemia symptoms, with telehealth-applicable as A, other chronic diseases as B, and nonchronic treatment as N. For special cases that do not fit in the preceding categories, we create an attribute, that is, Reim_Spe (*F*_*RS*_), to record these special telehealth applicable cases, with applicable denoted as Y and nontelehealth-applicable as N. Table 1 lists the attributes used to consult with the experts and generate the decision tree for each expert in the following discussion.

**Table 1:** Attributes Used to Consult with the Experts

Attributes	Data Type	Attributes	Data Type
Insurance Amount (*F*_*Ins_amt*_)	numeric	Gender	category
Age	numeric	Reim_Spe (*F*_*RS*_)	category
outpatient frequency (*F*_*op*_)	category	Economic Priority (*F*_*Eco*_)	category
inpatient frequency (*F*_*ip*_)	category	Age Group (*F*_*Age*_)	category
No. of outpatient times (op_time)	numeric	Insurance Level (*F*_*IL*_)	category
No. of inpatient times (ip_time)	numeric	Drug Duration (*F*_*DD*_)	category
No. of days in an emergency bed (EB day)	numeric	Chronic Bed Rate (*F*_*CBR*_)	numeric
No. of days in a chronic bed (CB day)	numeric	Target Disease (*F*_*TD*_)	category
No. of drug prescription days (drug day)	numeric	Copayment Exemption Mark (*F*_*CEM*_)	category
Remoteness (*F*_*R*_)	category	Distance (*F*_*Dis*_)	numeric
the amount of medical fees (Total amount)	numeric	Target Treatment (*F*_*TT*_)	category

The decision tree–building algorithm does not handle numeric attributes uniformly. When applying these attributes to generate the final decision tree, numeric attributes may be used more than once with different thresholds, and these numerical attributes should be transferred to categorical ones. First, for the numeric attributes that are already converted (e.g., age group), we remove the numeric attributes. Second, we can use mean and standard deviation to compute the thresholds and categorize the attributes (e.g., age). Table 2 presents the formula to convert numeric attributes into categorical ones. For example, distance is numeric; hence, we convert it into a categorical attribute *F*_*Dis_C*_ by using the formula in Table 2. Then, the converted attributes together with the categorical attributes in Table 1 are used to build the final decision tree classifier.

**Table 2:** Conversion Formula for Numeric Attributes in the Decision Tree Algorithm

Attribute	Conversion Formula
*F*_*Ins_amt_C*_	IF(*F*_*Ins_amt*_ < mean, 0, IF(*F*_*Ins_amt*_ < mean + standard deviation, 1, 2))
op_time_C	IF(op_time < mean, 0, IF(op_time < mean + standard deviation, 1, 2))
ip_time_C	IF(ip_time < mean, 0, 1)
EB day_C	IF(EB day < mean, 0, 1)
CB day_C	IF(CB day < mean, 0, 1)
drug day_C	IF(drug day < mean, 0, IF(drug day < mean + standard deviation, 1, 2))
Total amount_C	IF(Total amount < mean, 0, IF(Total amount < mean + standard deviation, 1, 2))
*F*_*CBR_C*_	IF(*F*_*CBR*_ < mean, 0, 1)
*F*_*Dis_C*_	IF(*F*_*Dis*_ < mean, 0, IF(*F*_*Dis*_ < mean + standard deviation, 1, 2))

#### Step 1.2 target variable generation

This study aims in classifying potential chronic patients who are suitable to receive telehealth services subsidized by health insurance reimbursements. This target variable does not exist in most healthcare data set. Thus, HDTTCA generates the target variable, that is, adoptability first. This approach involves asking experts to assign a value for the variable. However, because the data set is extremely large, HDTTCA samples a comparatively small data set as the training data set for experts’ opinions. To solve the problem of this study, we interview three experts in the telehealth-related fields (i.e., a physician, a social worker, and a manager of a care centers) in identifying the target variable, that is, adoptability (labeling the adoptability Y or N for each record) in the sampled data. Then, the experts reveal the criteria and rules they used in their decisions during the interview by showing all the attributes for all records in the sampled data in Table 2.

The experts label only a comparatively small data set. Therefore, HDTTCA generates a decision tree for each expert independently after collecting their opinions. The possible values of the target variable are “yes”, “no”, or “in consideration”. Given the importance of telehealth services in monitoring the physiological values and eliminating the urban–rural gap and socioeconomic gaps, we combine “no” and “in consideration” into one group. HDTTCA uses the attributes in Table 2 to generate the decision tree for each expert independently. Then, each decision tree is used to determine the value of the target variable for the entire data set. However, because expert opinions may not be consistent in some records, HDTTCA integrates the outcomes of each record by using the following rules: adoptability = Y if more than or equal to half of the experts labeled it as Y and adoptability = N if less than half of the experts labeled it as Y.

#### Step 1.3 data sampling and partitioning

Although patients suffering from chronic diabetes, hypertension, and heart diseases increase, our potential target population is still comparatively rare, accounting for 10% or less of the total population. These imbalance characteristic may reduce the predictability of a decision tree model; over- and undersampling are helpful techniques to overcome this problem. Oversampling resamples the existing minority data with slight modifications to be close to the proportion of majority, whereas undersampling abandons some existing majority data and keep all minority data. These techniques balance the data set distribution. We also adopt stratified sampling to increase the proportion of our target.

After sampling, we divided the data sets into training and validating subsets. We use the former to build the classification model and the latter in validating the overfitting problem and compare the prediction rate of different models. The overfitting problem occurs when the model used extremely many attributes to generate a decision tree and fit the data with extremely few objects. We may find overfitting clues in the model prediction step. If the decision tree’s accuracy is high in the training data set (i.e., due to the classifier’s objective in maximizing accuracy) but comparatively low in the validation data set, then an overfitting problem occurs. Therefore, we need to decide the proportion of training and validation data sets carefully. Given that HDTTCA samples a comparatively small data set similar to the training data set for experts’ opinions, we use all the experts’ opinions as the training data set and randomly took additional samples from the remaining data set as the validation data sets.

### Step 2: decision tree model building

In this step, we build a decision tree classifier, on the basis of the training data set and validate the classifier by using the validation data set. However, the telehealth service classification problem we consider the involved > 20 attributes, which are not all essential to identify the characteristics of the target variable. Correlations among the attributes can also cause multicollinearity and inaccuracy in some data-mining models. Thus, HDTTCA uses an attribute selection mechanism to choose the most discriminative attributes when building a decision tree classifier.

#### Step 2.1 attribute selection

Before detailing the construction of a classifier, we define *T* as the data set, which was constructed by *m* attributes and had *n* records, *x*_*1*_, *x*_*2*_, …, and *x*_*n*_. The target variable, which was denoted as *y*_*i*_ = {0, 1} where 1 ≤ *i* ≤ *n*, was “adoptability for telehealth service”. Therefore, a record can be expressed as *x*_*i*_ = [*x*_*i1*_, *x*_*i2*_, …, *x*_*im*_, *y*_*i*_] and *T* = {*x*_*i*_ | 1 ≤ *i* ≤ *n*}. Decision tree algorithms classify records by conjunctive rules (e.g. age group = elder and distance ≥60). Several decision tree algorithms, such as ID3 and C4.5, apply information theory to separate data by iteratively calculating the entropy, which was denoted as *H*(*T*) and the information gain, which was denoted as *IG*(*T*, *a*), from splitting data on the basis of the attribute *a*. Entropy, which was denoted as *H*(*T*), is the expected value of the information contained and can be defined as *H*(*T*) = −Σ*p*(*b*)log(*b*), where *T* is the training data set, *Y* is the target variable in *T*, *b* is a classified value in *Y*, and *p*(*b*) is the probability that an object in *T* is classified as *b*.

Information gain is the amount of uncertainty reduced due to the split, which can be defined as *IG*(*T, a*) = *H*(*T*) – Σ*p*(*a*)H(*a*), where *A* is an attribute for which a split has occurred, *p*(*a*) is the probability that an object in *T* exhibits attribute *A* = *a*, and *H*(*a*) is the entropy of the subset of *T*, where attribute *A* = *a*. The decision tree selects the locally best attribute (i.e., highest information gain) as a splitting criterion. After calculating the information gain of each attribute, the decision tree algorithm selects the attribute with the maximum information gain to be a node, which splits the data set into two or more subsets. The process iteratively proceeds until a full decision tree is built.

#### Step 2.2 decision tree classifier building

A well-fitted decision tree model can predict the training data set with the least misclassification cost or the highest accuracy. The advantages of decision trees over other classification algorithms are their coherent and consistent rules from the tree root to the leaves and the ease of interpretation. We use J48 in Weka [8], which is an open-source Java implementation of C4.5, as our decision tree–building algorithm. The C4.5 approach, which is used to calculate the difference in entropies among variables, is based upon the information gain of each attribute. The algorithm identified the attribute with the highest normalized information gain, which we choose as the splitting node.

When using J48 in Weka [8], we must determine two parameters, namely, the confidence factor and the minimum number of objects per leaf. The smaller the confidence factor is, the more pruning the algorithm will do. However, pruning reduces the accuracy of the training data while generally increasing the accuracy of unseen data. We use the confidence factor and the minimum number of objects per leaf in mitigating overfitting, where the decision tree will achieve perfect accuracy on training data, but the resulting decision tree is extremely specific that it does not apply to anything other than the training data. In general, if we reduce the confidence factor or increase the minimum numbers of objects per leaf, then the accuracy of the training set will decrease.

In Weka [8], J48 offers two settings to improve the estimation of sensitivity and accuracy, namely, training/test split and cross validation. However, the telehealth service classification problem needs input from human experts for the target variable, which leads to a small training data set and a large independent testing data set. In this case, neither option is appropriate for HDTTCA to adopt. We use different confidence factor levels and the minimum number of objects per leaf to discover the best location for the pruning confidence factor and the minimum number of objects per leaf, in which it prunes enough to make the learned decision tree sufficiently accurate on test data but does not sacrifice excess training data accuracy. The location where this spot of the pruning confidence factor and the minimum number of objects per leaf locate will depend upon the problem, and the only way to determine them reliably is performing an experiment.

An appropriate model should be able to predict future data sets consistently and effectively. Different perspectives and criteria are available to identify the performance across different settings of the confidence factor and the minimum number of objects per leaf. The validation techniques in J48 in Weka can assess the performances of various settings. Considering that the training data set is small, HDTTCA will not split or cross-validate the training set. Instead, HDTTCA asks J48 in Weka to randomly produce 30 data sets from the test data set and apply validation and evaluation on these data sets. If the error between the training and validation data is high, then overfitting or underfitting needs to be considered.

### Model assessment

Then, we compared the final decision tree results against the results obtained from a logistic regression model that was constructed by Weka [8]. We first compute the Spearman coefficients of correlation (*r*_*s*_) among these attributes and eliminate the coefficient with *r*_*s*_ > .7 to avoid the multicollinearity problem. Then, we perform stepwise logistic regression to select the significant attributes from the remaining attributes. We remove unrelated attributes according to coefficient tests or *p*-value greater than 5%, until all remaining attributes are significant.

Finally, the predictive capability of the decision tree is a potential issue. HDTTCA compares the prediction, which is denoted as *ŷ*, with the actual result of the target variable, *y* to test for predictive capability. Table 3 demonstrates different classification results. Some common measures in selecting the best decision tree classifier are the misclassification rate, which is denoted as *Err*(*T*), and the probability of being correct, which is denoted as *Accuracy*(*T*). Here, *Err*(*T*) is computed as (*FP* + *FN*) / (*FP* + *TN* + *TP* + *FN*), and *Accuracy*(*T*) is computed as 1 – *Err*(*T*) or (*TP* + *TN*) / (*FP* + *TN* + *TP* + *FN*), where *FP* is the false positive count, *FN* indicates the false negative count, *TP* is the true positive count, and *TN* reflects the true negative count (Table 3).

**Table 3:** Different Classification Results

Predicted / Actual	Actual Positive (*y*_*i*_ = 1)	Actual Negative (*y*_*i*_ = 0)
Predicted as Positive (${\hat{y}}_i=1$)	True Positive (*TP*)	False Positive (*FP*)
Predicted as Negative (${\hat{y}}_i=0$)	False Negative (*FN*)	True Negative (*TN*)

Another important criterion is sensitivity or true positive rate, which is denoted as *Sensitivity*(*T*), that can be computed as *TP*/(*TP* + *FN*), such that the denominator represents actual positive cases. Sensitivity can identify the positive case of a model correctly. Hence, high sensitivity implies that few Type-II errors have occurred when applying the model. Low sensitivity suggests a poor performance in identifying the wrong patients for telehealth services. Specificity, which is computed as *Specificity*(*T*) = *TN*/(*FP* + *TN*) = 1 – *FP*/(*FP* + *TN*), is the true negative rate and indicated how accurately our model will identify true negatives. Precision, which is computed as *Precision*(*T*) = *TP*/(*TP* + *FP*) = 1 – *FP*/(*TP* + *FP*), is the exactness or percentage of tuples that the classifier labeled as positive that are actually positive. Precision denoted the accuracy of our model in identifying true positives.

In general, the misclassification rate or *Accuracy*(*T*) evaluates classification models. However, *Accuracy*(*T*) cannot distinguish type-I/type-II error. For the telehealth service classification problem in this study, type-II error is crucial. Patients can suffer extensively if the model misidentified an eligible patient as ineligible. Therefore, sensitivity is the first criterion that we will apply in the evaluation process, followed by accuracy, specificity and precision.

## Results

### Real-world health insurance research data set

We acquired a data set from the NHIRD provided by the NHIA, Ministry of Health and Welfare in Taiwan, which contains data from 1996, when Taiwan first introduced NHI, to 2012 in demonstrating the applicability of HDTTCA. For calculative convenience, we use the 2012 data as our research scope, which consists of 55,389 distinct hospitals and 653,209 distinct patients with 15,882,153 outpatient and 135,775 inpatient records. After HDTTCA’s generating derivative attribute step, the size of the data set decreases to approximately 100 MB compared with the 4.45 GB size of the original data set. This study (REC no: 20141HS007) has been approved by the Research Ethics Committee of National Taiwan University and classified as exempt on November 14, 2014 in accordance with the Social and Behavioral Research Ethical Principles and Regulation of National Taiwan University and governmental laws and regulation of Taiwan.

After Step 1.1, HDTTCA has derived 22 attributes (Table 1). Table 4 shows the distributions of the nine telehealth-related attributes. Approximately 91% of the patients live in nonremote area, and 98% of the patients are not socioeconomically disadvantaged, which indicates that they possessed considerable access to medical resources. Over 91% of the patients have the target diseases that are suitable for telehealth services.

**Table 4:** Distribution of the Attributes Used in Oversampling

Attributes	Distribution of each class		
Remoteness (*F*_*R*_)	1	2	3
	90.59%	7.32%	2.1%
Economic Priority (*F*_*Eco*_)	N	Y	
	97.6%	2.4%	
Age Group (*F*_*Age*_)	Elder	Middle-aged	Young
	9.72%	79.86%	10.42%
Insurance Level (*F*_*IL*_)	High	Middle	Low
	24.74%	55.86%	19.4%
Drug Duration (*F*_*DD*_)	N	Y	
	87.76%	12.24%	
Chronic Bed Rate (*F*_*CBM*_)	= 0	> 0	
	99.84%	0.16%	
Target Disease (*F*_*TD*_)	N	Y	
	91.38%	8.62%	
Target Treatment (*F*_*TT*_)	N	Y	
	99.998%	0.002%	
Copayment Exemption Mark (*F*_*CEM*_)	N	Y	
	95.28%	4.72%	

To ensure that the sampling training data set is closely representative of the actual patients’ distribution, we use stratified sampling on the basis of two basic attributes, that is, gender and age group. However, the nature of our problem indicated possible unbalanced distribution of the target variable is. Therefore, sufficient telehealth-applicable patients should be sampled into the training data set. Therefore, we adopt the oversampling technique on the basis of telehealth-related attributes. We obtain 200 samples out of 653,209 records. Table 5 shows the patients’ distributions of gender and age groups, and Table 6 shows the distribution in the sample.

**Table 5:** Distribution of Gender and Age Group of All Patients in the entire Data Set

Attributes	Age Group			
Gender	Elder	Middle-aged	Young	Percentage
Female	4.45%	38.87%	5.82%	49.14%
Male	5.28%	40.99%	4.59%	50.86%
Percentage	9.72%	79.86%	10.42%

**Table 6:** Distribution of Gender and Age Group of All Patients in the Sample Data Set

Attributes	Age Group			
Gender	Elder	Middle-aged	Young	Percentage
Female	10%	28%	5.5%	43.5%
Male	8%	40.5%	8%	56.5%
Percentage	18%	68.5%	13.5%	

Given that, elderly is more likely to be eligible for telehealth services, the sample size of elderly patients is twofold larger than those of other samples. HDTTCA also used other attributes, such as remoteness and economic priority (Table 4) to perform oversampling. The sample contained more male patients than female ones because the target diseases are more common in male population than in female ones.

### Generating a decision tree for each expert and final target variable

In this part, we describe our interviews with three experts in telehealth-related fields, namely, a physician in a medical center as Expert 1, a social worker in a remote area as Expert 2, and a manager in a long-term care center as Expert 3, to identify the target variable of adoptability in the sample data set. During each interview, we first spend 10 min to introduce our research objective and gave each expert an outline of the data set. Afterward, each expert used 30 min to label the adoptability of each record with Y or N. Finally, the experts explained the criteria they used to make their decisions. Then, we use adoptability as the criteria from each expert to generate the decision tree. Given that the sample size of the training data set is only 200 records, we set the minimum number of records per leaf to be 1, which indicates that it contained 0.5% of the data set. Then, using J48 in Weka as the decision tree-building algorithm, we build the decision tree for each expert and discuss the performance and the rules of each decision tree. Finally, we generate the final target variable by using these decision trees for the testing data set.

Expert 1 focuses on the clinical record, such as the target diseases, CB days and the frequency of inpatient admission. Therefore, the first few branches in the decision tree are all related to the clinical attributes. The physician also indicates that telehealth services can reduce the time in the hospital after the equipment detected abnormal values. The service provided a standard value for patients to reduce unexpected outpatient times.

**Fig. 2:**
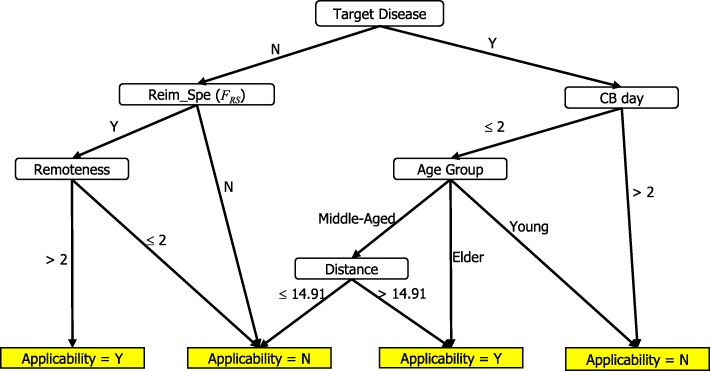
Physician’s Decision Tree

Table 7 shows the classification outcome and statistics of Expert 1’s version. Figure 2 shows the full decision tree of Expert 1’s version, which possessed eight leaves with a tree size of 14 and a depth of four levels. For example, one rule stated that if a middle-aged patient has a target disease has been hospitalized chronically in bed for > 2 days and lives > 14.91 km away from the hospital, then the patient is qualified for telehealth services reimbursed by the medical insurance policy.

**Table 7:** Results of Physician’s Decision Tree on Training Data

	Classified as		
Actual	Y	N	
Y	14	9	
N	3	174	
Sensitivity: 60.9%	Specificity: 98.31%	Precision: 82.35%	Accuracy: 94%

Expert 2 focuses on welfare-related attributes, such as copayment exemptions and patients’ residence. Expert 2 thinks that telehealth services can reduce the urban-rural gap and improve the life of rural residents.

**Fig. 3:**
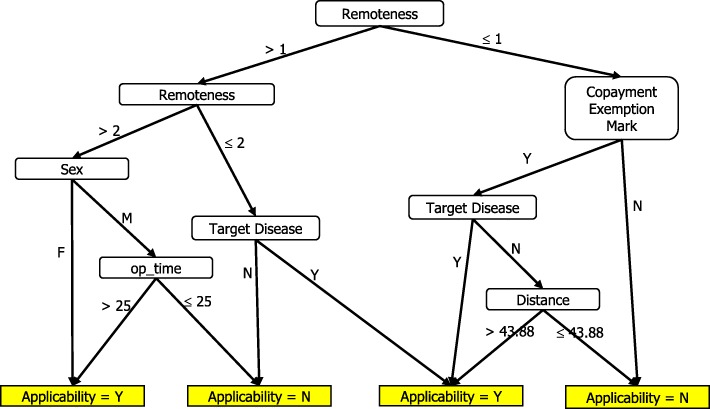
Social Worker’s Decision Tree

Table 8 shows the classification outcome and statistics of Expert 2’s version. Figure 3 shows the full decision tree, which consisted of 9 leaves with a tree size of 17 and a depth of 4 levels. For example, one rule states that if a copayment-exempted patient that lives in a rural area does not have target diseases and has a smaller than or equal to 43.88 km traveling distance between his residence and hospital, then the patient is unqualified for telehealth service reimbursed by the medical insurance policy.

**Table 8:** Results of Social Worker’s Decision Tree on Training Data

	Classified as		
Actual	Y	N	
Y	16	4	
N	4	176	
Sensitivity: 80%	Specificity: 97.78%	Precision: 80%	Accuracy: 96%

Expert 3 emphasizes convenience and accessibility of healthcare for patients and also encourages elderly patients to use telehealth equipment in her long-term care center frequently. Therefore, the important attributes for Expert 3 are distance and age.

**Fig. 4:**
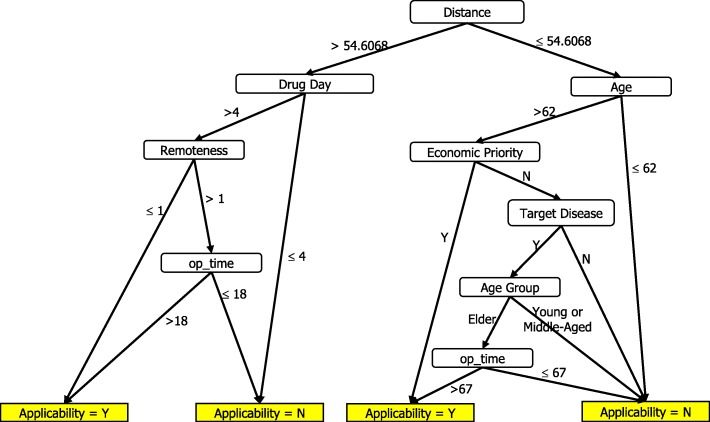
Manager’s Decision Tree

Table 9 shows the classification outcome and statistics of Expert 3’s version, and Fig. 4 shows her full decision tree, which contains 11 leaves with a tree size of 20 and a depth of six levels. For example, one rule states that if a patient lives in a rural area, has more than 4 days of drug prescription, has more than 18 days of outpatient time, and has a > 54.61-km traveling distance between his residence and hospital, then the patient is qualified for telehealth service reimbursed by the medical insurance policy.

**Table 9:** Results of Manager’s Decision Tree on Training Data

	Classified as		
Actual	Y	N	
Y	18	4	
N	1	177	
Sensitivity: 81.81%	Specificity: 99.44%	Precision: 94.74%	Accuracy: 97.5%

Considering that the opinions from the three experts are inconsistent, we need to generate the final value of the target variable for each record in the training data set using the following rules: labeling adoptability as Y if two or more experts indicate its eligibility and N if one or no expert says that it is eligible. Table 10 shows the outcome distribution in each expert’s version and the final version.

**Table 10:** Distributions of Adoptability in Different Versions of the Sample Data Set

Adoptability	Expert 1	Expert 2	Expert 3	Final Version
Applicable or “Y”	23	20	22	14
Not Applicable or “N”	177	180	178	186

For the testing data set, we generate the opinions of each expert for each record by using the decision tree of each expert generated in Figs. 2, 3 and 4. The three experts’ opinions are inconsistent, and we need to generate the final value of each record in the testing data set by using the same rules. After obtaining the target variable, we are ready to create a decision tree of the final version.

### Building the final version decision tree and logistic regression model

Given that the HDTTCA samples a comparatively small data set (200 patients) for experts’ opinions, HDTTCA used all experts’ opinions as the training set and took 30 random samples of 20,000 patients each from the remaining data set as the validation data sets when training the decision tree. When using J48 in Weka [8], two parameters, such as the confidence factor and the minimum number of objects per leaf, need to be determined. We use six different settings of the confidence factor and the minimum number of objects per leaf, (i.e., 0.25, 0.5, and 0.75) versus (1, 2) in discovering where it prunes enough to make the learned decision tree sufficiently accurate on testing the data but does not sacrifice excess accuracy on the training data.

In total, J48 in Weka [8] generates six trees for the six different settings of the confidence factor and the minimum number of objects per leaf. However, tree (0.25, 1) is the same as tree (0.25, 2) and tree (0.5, 1) is the same as tree (0.5, 2). Therefore, we compare the performance metrics of the 30 testing data sets for the four trees by using ANOVA, as shown in Table 11. The ANOVA results reject all null hypotheses for the four metrics. We also perform a pairwise *t*-test to compare the top two trees, that is, (.25, 1 or 2) and (.75, 2), and the *t* statistics and *p*-value for the sensitivity metric are 19.0614 and 3.01E-18, respectively, which rejects the null hypothesis. Thus, we can select the best trees by using the sensitivity criterion, which is tree (.75, 2).

**Table 11:** Performance Metrics and ANOVA Tests for the Four Trees

Tree	Sensitivity	ANOVA	Accuracy	ANOVA	Specificity	ANOVA	Precision	ANOVA
(0.5, 1 or 2)	.7562	F = 8773.99	.9536	F = 17,067.17	.9656	F = 17,232.80	.5863	F = 13,619.86
(0.25, 1 or 2)	.9796		.9874		.9879		.8380	
(0.75, 2)	.9877	p_value = 7.8999E–108	.9626	p_value = 2.282E–120	.9610	p_value = 1.5E–120	.6176	p_value = 4.0981E–116
(0.75, 1)	.8836		.9442		.9481		.5201	

**Fig. 5:**
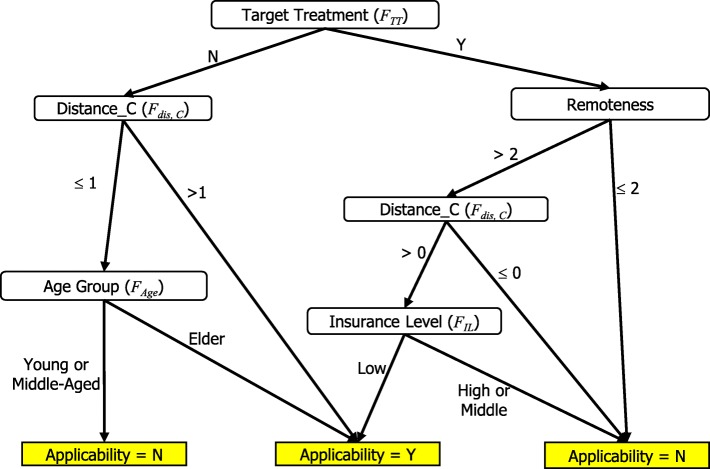
The Final Version

Table 12 shows the classification outcome and statistics of the final tree version (.75, 2) and Fig. 5 shows the full decision tree, which included 9 leaves with a tree size of 15 and a depth of 4 levels. For example, one rule states that if a patient lives in a rural area, has a traveling distance greater than the population mean (i.e., 21 km), has no target diseases, and has a middle-level insurance policy, then the patient is unqualified for telehealth service reimbursed by the medical insurance policy.

**Table 12:** Results of the Final Version (0.75, 2) Decision Tree on Training Data

	Classified as		
Actual	Y	N	
Y	11	2	
N	1	186	
Sensitivity: 91.17%	Specificity: 98.94%	Precision: 84.62%	Accuracy: 98.5%

In conclusion, three main dimensions influenced the decision about using telehealth services. First, patients’ clinical status is important because of the limitation of telehealth equipment. Patients with diseases that do not need to be monitored by physiological values are not recommended for telehealth services. Second, telehealth services play important roles for patients who live in inconvenient areas or who have long travel times to healthcare facilities. Third, telehealth service benefits elderly and low socioeconomic level patients. With telehealth service subsidized by insurance, these patients can have a healthy quality of life.

After HDTTCA produces the final version of decision tree, the rules can be used to assign the values of the target variables in the entire NHIRD. Our data indicate that 3.56% (23,262 out of 653,209) of the patients are eligible for telehealth services in 2012. The following step is setting up different cases of experiments to compare the results of HDTTCA with those of a logistic regression model.

Then, we then construct the logistic regression model by Weka [8] as the baseline comparison model to the final decision tree. Before generating the logistic regression model, we need to determine the subset of attributes in Tables 1 and 2 that are suitable for the model. We compute the *r*_*s*_ among these attributes and eliminate the coefficient with *r*_*s*_ > .7 to avoid the multicollinearity problem. We perform stepwise logistic regression to select significant attributes from the remaining 13 attributes. The final logistic regression model consists of only 5 significant attributes (*p*-value ≥5%), as shown in Table 13. The coefficients of the five attributes are all positive, which indicates that the patients traveling a long distance toward the hospital, living in remote areas in an elderly age group, with copayment exemption mark, and those with target diseases are likely to qualify for the insurance reimbursements of telehealth services. Table 14 shows the classification of the outcomes and statistics of the final logistic regression model.

**Table 13:** Logistic Regression Model

Coefficients	Estimate	Std. Error	z value	Pr(>|z|)	Signif. codes
(Intercept)	−17.949	4.6122	−3.892	9.95E–05	***
Distance_C (*F*_*dis_C*_)	3.033	.9792	3.098	.001952	**
Remoteness (*F*_*R*_)	2.5578	1.1479	2.228	.025866	*
Age Group (*F*_*Age*_)	3.5081	1.2511	2.804	.005048	**
Copayment Exemption Mark (*F*_*CEM*_)	3.8109	1.5376	2.479	.013192	*
Target Disease (*F*_*TD*_)	8.0175	2.2871	3.506	.000456	***

Ps. Signif. codes: ‘***’ for 0; ‘**’ for 0.001; ‘*’ for 0.01

**Table 14:** Logistic Regression Model on the Training Data

	Classified as		
Actual	Y	N	
Y	10	2	
N	4	184	
Sensitivity: 83.33%	Specificity: 97.87%	Precision: 71.43%	Accuracy: 97.00%

## Discussion

This study proposes HDTTCA to determine the eligibility for the insurance reimbursements of telehealth services. After finding the feasible combinations of factors, models, and corresponding parameters in the Method Section, we conduct a series of experiments 30 times to compare the HDTTCA results with the results of the logistic regression by measuring their average performances and determining which model addresses the telehealth patient classification problem better. As mentioned in the Method section, four important metrics including sensitivity, accuracy, specificity, and precision, are used to compare the results. These metrics reflect the usability and accuracy of a model. We also discuss the interpretability of the result as a crucial criterion when applying different classification methods in practice.

Then, we then perform the experiments 30 times by taking 30 random samples of 20,000 patients each from the remaining data, and we measure their average performances. We set the decision tree of HDTTCA and the previously mentioned logistic regression model in Weka [8], which automatically searches for the final solution for each sample in the testing data set. To compare the results of the two models, we conduct pairwise *t*-tests between the results classified by the decision tree of HDTTCA (Table 12) and those classified by the logistic regression model (Table 13).

Given that this data set is unbalanced (only 3.56% of the patients are eligible for the telehealth services), HDTTCA result shows extremely high sensitivity, accuracy, and specificity (all > .95%) but low precision. The results in Table 15 reveal the average performances, and corresponding variances and *p*-values, that is, *P* (*T < =r*), of the pairwise *t*-tests between the decision tree generated by HDTTCA and the logistic regression model. In terms of sensitivity, the decision tree generated by HDTTCA and the logistic regression model are on the equal ground. In terms of accuracy, specificity, and precision, the decision tree generated by HDTTCA provides a better performance than that of the logistic regression model. The decision tree model generates a competitive performance and provides clear, easily understandable rules by applying HDTTCA. Hence, HDTTCA is a suitable choice in solving telehealth service classification problems.

**Table 15:** Pairwise *t*-tests for Performance Metrics

Metric	Sensitivity		Accuracy		Specificity		Precision	
Model	HDTTCA	LR	HDTTCA	LR	HDTTCA	LR	HDTTCA	LR
Mean	.9877	.9875	.9626	.9451	.9610	.9424	.6176	.5219
Variance	9.2187E–06	6.4638E–06	1.6201E–06	3.2124E–06	2.1E–06	3.856E–06	.00012	.0001293
DF	29		29		29		29	
*t*	−.7327		−81.5205		−79.4559		−88.2711	
P(T < =r)	.2348		4.1444E–36		8.69E–36		4.16E–37	

## Conclusion

In conclusion, this study has three contributions. The first contribution is confirming that the use of decision trees is a good approach in identifying the potential receivers of telehealth services. A decision tree telehealth service classifier can produce clear and understandable rules within extremely fast training time by applying HDTTCA. The second contribution indicates that HDTTCA determines the three most important dimensions of reimbursing patient for telehealth services, namely, clinical records, convenience, and social-economic status. The third contribution is proving that HDTTCA is essentially applicable on a real data set from NHIRD in Taiwan.

Two matters need to be illustrated in this study. First, the ethical question of denying some people access to a service because it is not covered by insurance or they live extremely near the hospital or service provider has not been addressed. That limitation indicates that the universal healthcare coverage is not universal if some cannot access it. Second, HDTTCA involves human judgment to determine the target variable. In the future, the actual value of the target variable may be acquired from the NHIRD if the policy of reimbursing patients for telehealth services is implemented. We can compare the HDTTCA results and the attributes of actual applicants and modify the classifier rules. Future work can also focus on building the utility model of users and in designing an appropriate billing mechanism of telehealth services.

## Data Availability

National Health Insurance Research Database of Taiwan contacting protected health information is not available to share.

## Abbreviations

CB: Chronic bed
EB: Emergency bed
HDTTCA: Heuristic decision tree telehealth classification approach
NHI: National Health Insurance
NHIA: National Health Insurance Administration
NHIRD: National Health Insurance Research Database
